# Intra-Articular Injection of Injectable Platelet-Rich Fibrin (i-PRF) for the Management of Internal Derangement of the Temporomandibular Joint: A Case Series

**DOI:** 10.7759/cureus.110512

**Published:** 2026-06-09

**Authors:** Satheesh G, Pratebha Balu, James Antony Bhagat, Ajay Kumar Jagdish, Selvakumar R

**Affiliations:** 1 Oral and Maxillofacial Surgery, Adhiparasakthi Dental College and Hospital, Melmaruvathur, IND; 2 Research, Sri Balaji Vidyapeeth (Deemed to be University), Indira Gandhi Institute of Dental Sciences, Puducherry, IND; 3 Periodontology, Sri Balaji Vidyapeeth (Deemed to be University), Indira Gandhi Institute of Dental Sciences, Puducherry, IND; 4 Oral and Maxillofacial Surgery, Asan Memorial Dental College and Hospital, Chengalpattu, IND

**Keywords:** arthrocentesis, clicking sounds, injectable platelet rich fibrin (i-prf), internal derangement of temporomandibular joint, tmj pain

## Abstract

Internal derangement (ID) of the temporomandibular joint (TMJ) is a common condition that is characterized by an abnormal relationship between the articulating disc and the mandibular condyle, often resulting in pain during jaw movements, joint sounds, and restricted mouth opening. Etiology of ID of TMJ is often multifactorial, involving functional, mechanical and biological factors that disturb the normal relationship between the disc and the condyle. Management strategies range widely from conservative therapy, minimally invasive procedures, to invasive surgical procedures. Arthrocentesis is a procedure that is minimally invasive, involving lavage of the superior joint space of the TMJ using needles to remove inflammatory mediators and release adhesions of the disc to the mandibular fossa. Also, there is an increasing interest in minimally invasive regenerative approaches that can heal the injured disc and attachments of the articular disc. Injectable platelet-rich fibrin (i-PRF), which is an autologous blood product, is rich in growth factors and has shown very promising results in reducing inflammation and promoting tissue repair. i-PRF can easily be produced by centrifuging autologous blood at 700 rpm for a very short time of 3 minutes. This case series evaluates the clinical outcomes of arthrocentesis followed by intra-articular i-PRF injections into the upper joint space in three patients diagnosed with TMJ internal derangement. All three patients presented with pain, limited mouth opening, and joint sounds. MRI investigation was done, and found one patient with an anteriorly displacing disc with reduction during opening and closing the mouth, and two patients with an anteriorly displacing disc without the disc reducing during opening and closing the mouth. Clinical parameters like intensity of pain measured by the visual analogue scale (VAS), joint sounds, maximum mouth opening (MMO), functional limitation and patient satisfaction were assessed preoperatively and during follow-up at one week, one month and three months postoperatively. The results demonstrated a significant reduction in pain scores, with mean VAS decreasing from 5.67 preoperatively to 0 at three months post-treatment. Mean maximum mouth opening improved from 32 mm to 39.3 mm, and joint sounds were eliminated in all cases at three months post-intervention. No complications or adverse effects were observed during and after the procedure. All three patients were satisfied with the treatment outcome. These findings suggest that arthrocentesis, which is followed by intra-articular injection of i-PRF, is a safer and effective treatment option for TMJ internal derangement, providing significant symptomatic relief and functional improvement. Further studies with larger sample sizes, post-intervention MRI scan evaluation after three months and longer follow-up periods were recommended to validate these outcomes.

## Introduction

Internal derangement (ID) of the temporomandibular joint (TMJ) represents a group of disorders that are characterized by an abnormal relationship between the mandibular condyle, the articular disc and articular eminence [1]. It is one of the most common problems, causing orofacial pain and dysfunction, frequently presenting with joint pain, clicking or crepitus, and limitation of mandibular movements like opening, closing and lateral movements [2]. The condition is multifactorial in origin, with contributing factors including trauma, parafunctional habits, occlusal discrepancies, and degenerative changes within the joint [3,4]. ID can be broadly classified into anterior disc displacement with reduction or without reduction based on the clinical and radiological findings. MRI is the preferred imaging modality to view the disc and its attachments. Wilkes classification system classifies this condition into five stages, ranging from early, painless disk displacement to advanced degenerative joint disease [5,6]. Management of TMJ internal derangement typically follows a stepwise approach, beginning with conservative modalities such as patient education, occlusal splints, physiotherapy, and pharmacotherapy. While many patients respond favourably to these measures, some continue to experience persistent symptoms, necessitating minimally invasive or surgical interventions [1,7]. Arthrocentesis and arthroscopy have been widely used to lavage the joint, reduce intra-articular inflammation, and improve joint mobility [8]. However, these techniques primarily address the mechanical and inflammatory components and may not directly promote tissue regeneration. In recent years, regenerative medicine has gained significant attention in the management of musculoskeletal disorders, including TMJ pathologies. Injectable platelet-rich fibrin (i-PRF), a second-generation platelet concentrate obtained through low-speed centrifugation (700 rpm for 3 minutes), has emerged as a promising biologic adjunct. Unlike earlier platelet concentrates, i-PRF exists in a liquid form that allows for easy intra-articular delivery and provides a useful sustained release of many growth factors such as platelet-derived growth factor, transforming growth factor-beta, and vascular endothelial growth factor [5,6]. These bioactive molecules play a crucial role in tissue repair, angiogenesis, and modulation of inflammation, thereby offering potential therapeutic benefits in TMJ disorders. The application of i-PRF in TMJ internal derangement is relatively novel, with limited clinical data available in the literature. Its autologous nature, ease of preparation, and minimal risk profile make it an attractive alternative or adjunct to conventional treatment modalities [9-11]. The aim of this case series is to evaluate the clinical outcomes of arthrocentesis followed by intra-articular i-PRF injections in patients with TMJ internal derangement, focusing on pain reduction, improvement in mouth opening, restoration of joint function and patient satisfaction level.

## Case presentation

Case 1

**Figure 1:**
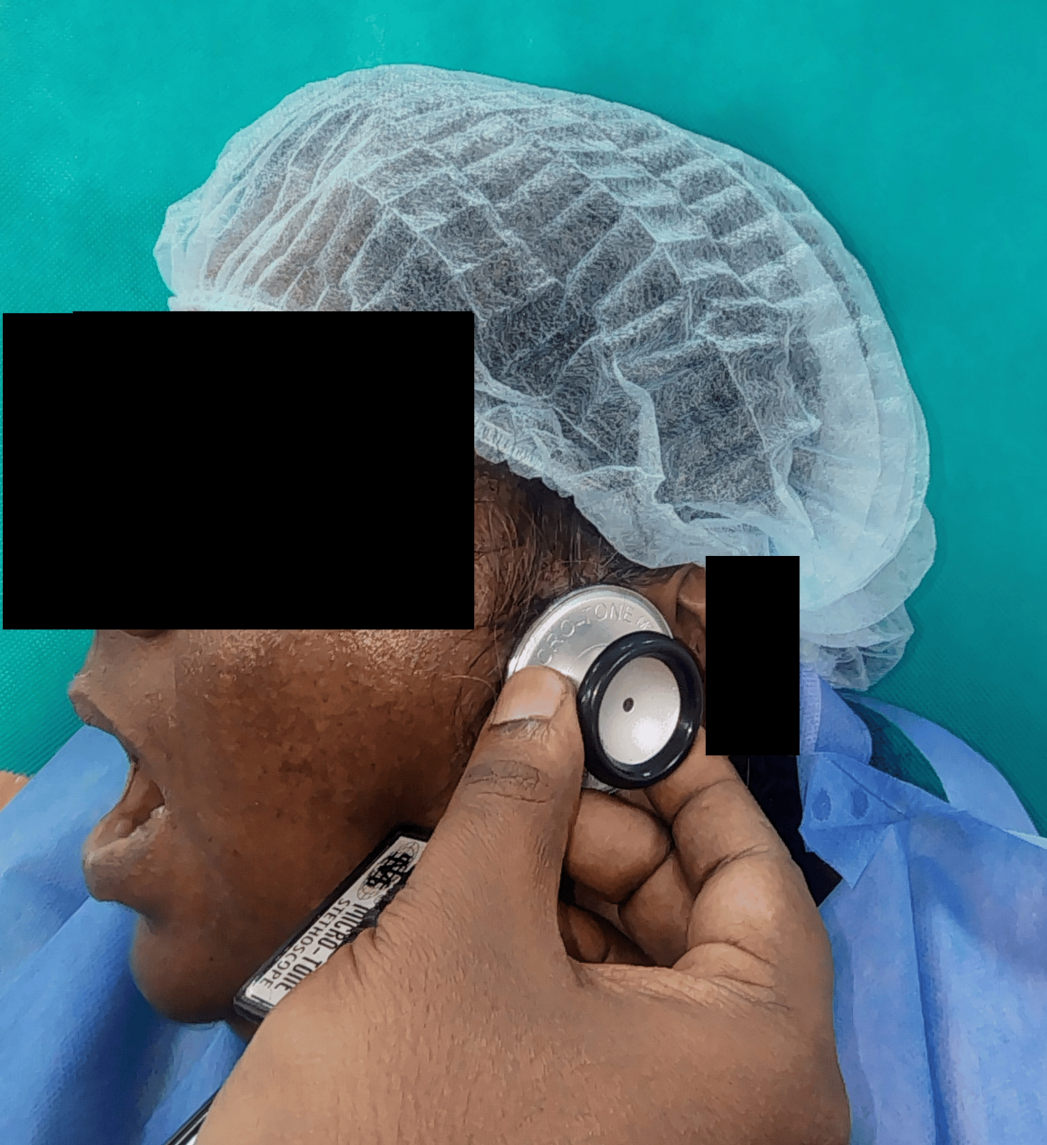
Auscultation for joint sounds

**Figure 2:**
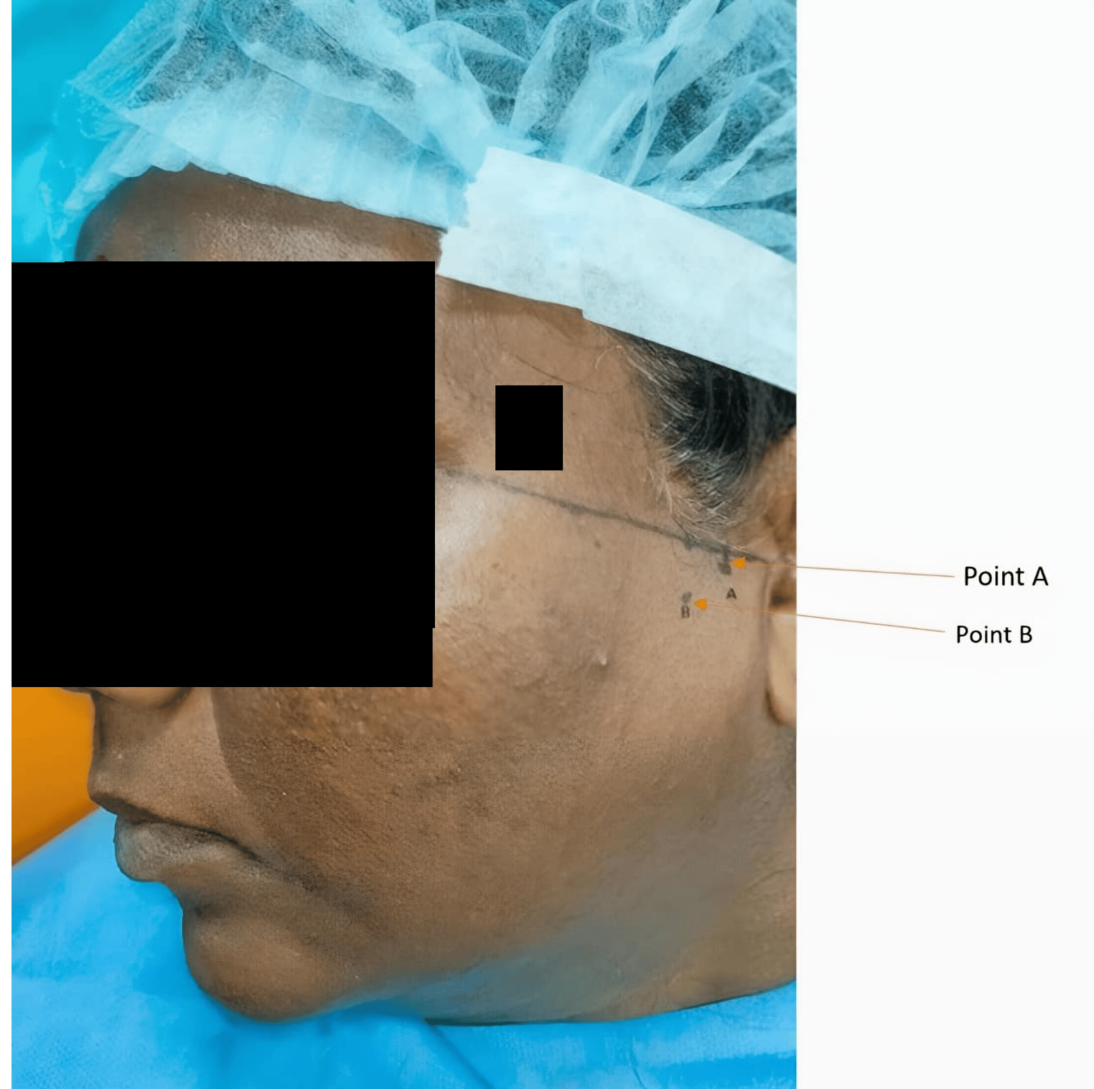
Marking for arthrocentesis using 2-needle technique. Point A, 10 mm anterior to mid tragal point and 2 mm inferior; Point B, 10 mm anterior to point A along the line drawn from mid tragal point to outer canthus of eye and 10 mm inferior to point A.

**Figure 3:**
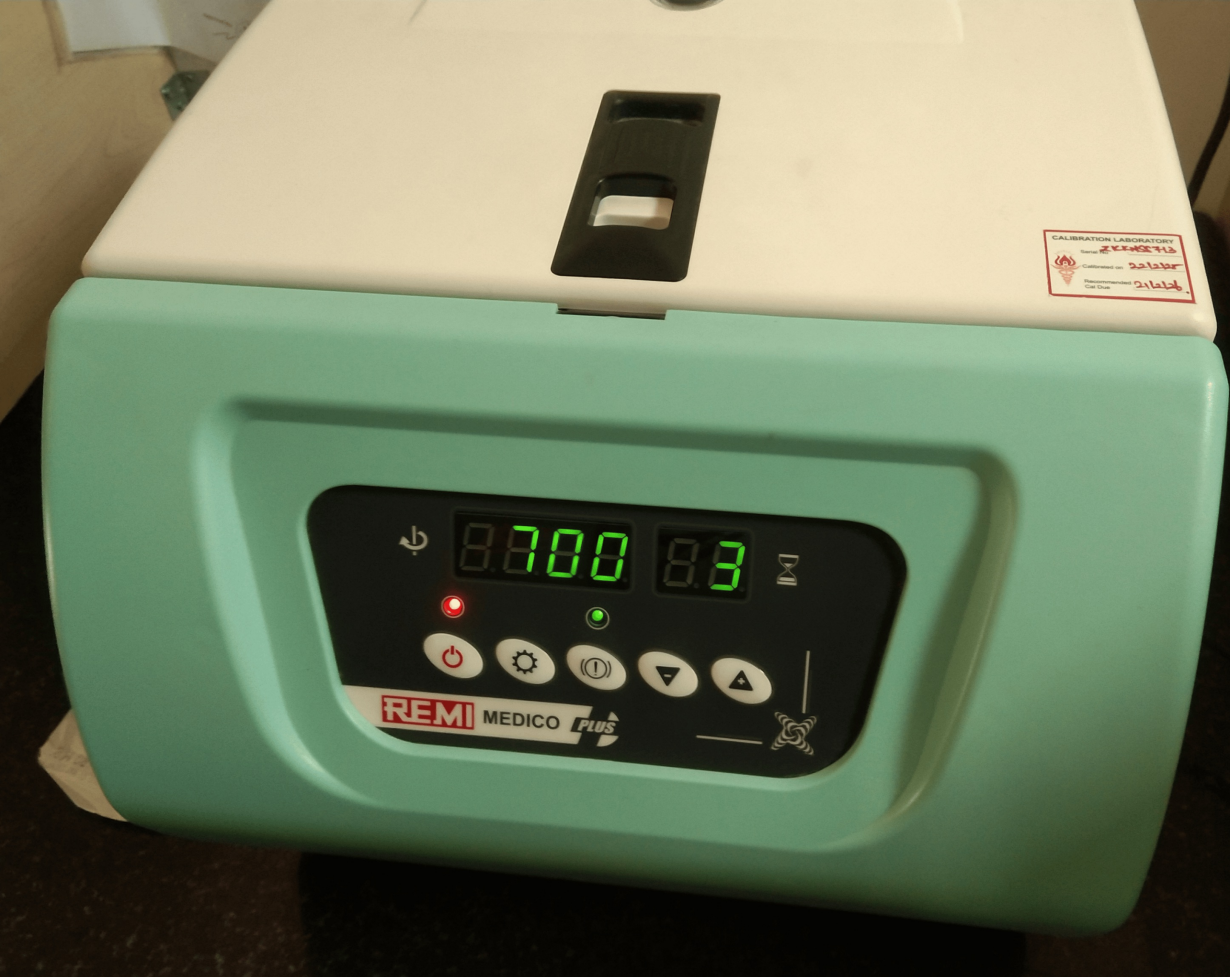
Centrifuge machine (Remi Medico plus) for i-PRF generation with setting of 700 rpm and 3 minutes time

**Figure 4:**
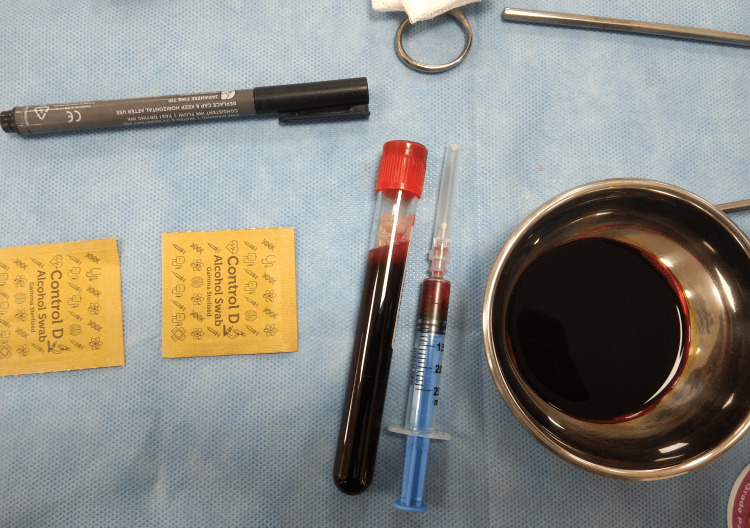
Marking pen, alcohol swab for surgical site disinfection and the prepared i-PRF

**Figure 5:**
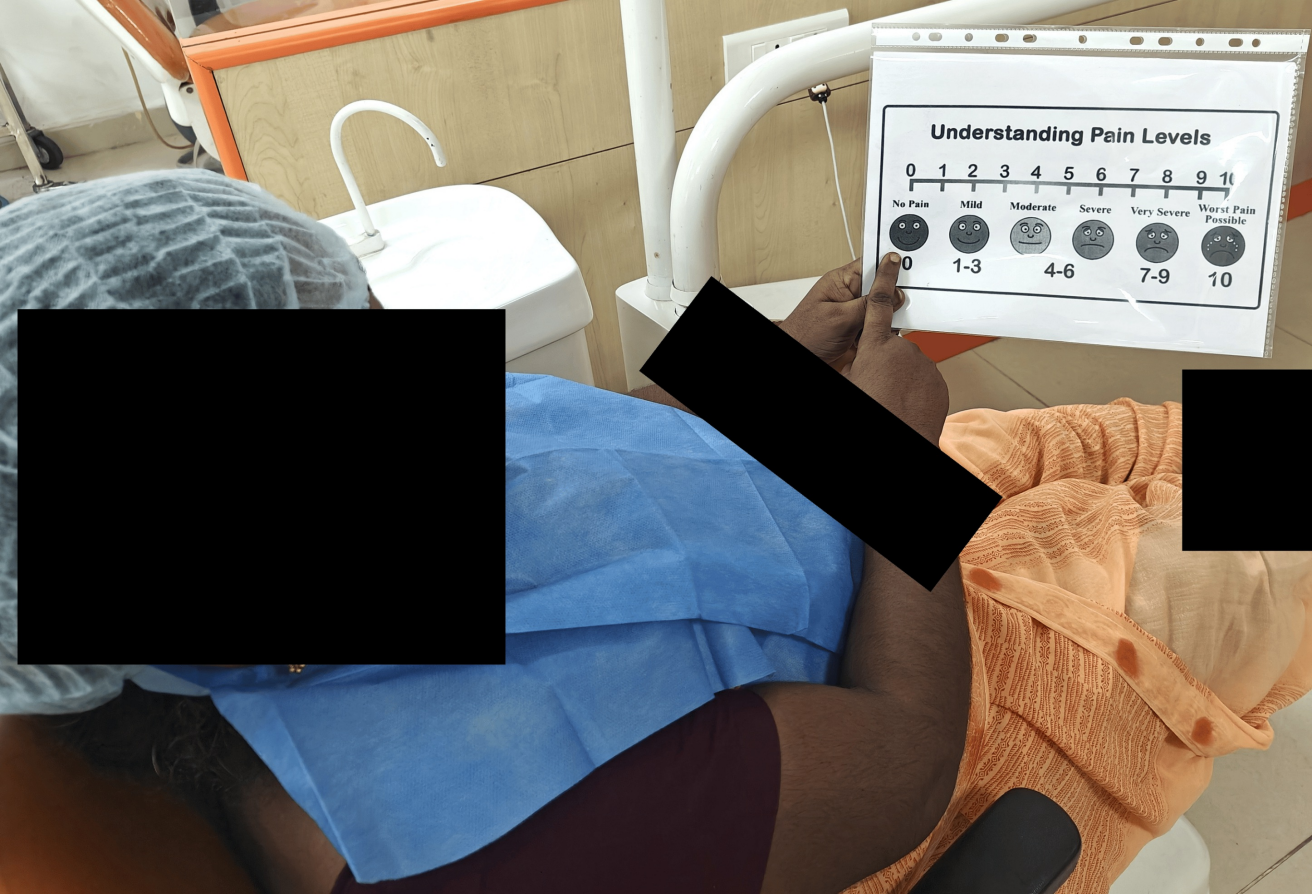
The patient showing a VAS scale of score 0 post-intervention after follow-up of three months.

A female patient aged 42 years presented to the department of oral and maxillofacial surgery, Adhiparasakthi Dental College and Hospital (APDCH), Melmaruvathur, with a complaint of pain over the left temporomandibular joint region (TMJ) and limited mouth opening for the past two months. Her pain got aggravated while chewing food. She had the same complaint three months ago, for which she was advised a soft diet, hot fomentation over the right TMJ region, to limit excessive mouth opening, and was advised to wear an occlusal splint. She had pain relief for one month, after which she again developed pain over the left TMJ region and limited mouth opening. On examination, the maximum mouth opening was 28 mm, and the mouth deviates to the left during mouth opening. Auscultation for TMJ joint sounds was done as shown in Figure 1. Intraoral examination revealed Angle's class I malocclusion, with no missing teeth. On palpation, tenderness was present over the temporalis muscle, and on auscultation, clicking was present during opening the mouth on the left side. MRI investigation was done, and it revealed a non-reducing articular disc during opening and closing the mouth, suggestive of Wilkes stage III classification of internal derangement of the left temporomandibular joint. The treatment plan was to do arthrocentesis followed by i-PRF injection into the left TMJ. Arthrocentesis was performed using a 2-needle technique (point A and point B) according to Nitzan guidelines, as shown in Figure 2, using 100 ml of Ringer’s lactate solution. i-PRF was prepared from 10 ml of autologous blood collection and centrifugation using a low-speed centrifugation protocol (700 rpm for 3 min). Figure 3 shows the centrifuge machine (Remi Medico plus, Remi, Mumbai, India) for i-PRF generation with a setting of 700 rpm and 3 minutes. The obtained i-PRF, as shown in Figure 4, was injected into the upper joint space of the TMJ through the needle in the point A position. The patient was evaluated for pain, joint sounds, mouth opening, functional limitations like chewing, yawning, speech, and patient satisfaction level at one week, one month and three months post-intervention. All the values are tabulated as shown in Table 1. The patient showed a VAS score of 0 post-intervention after follow-up of three months, as shown in Figure 5.

Case 2

**Figure 6:**
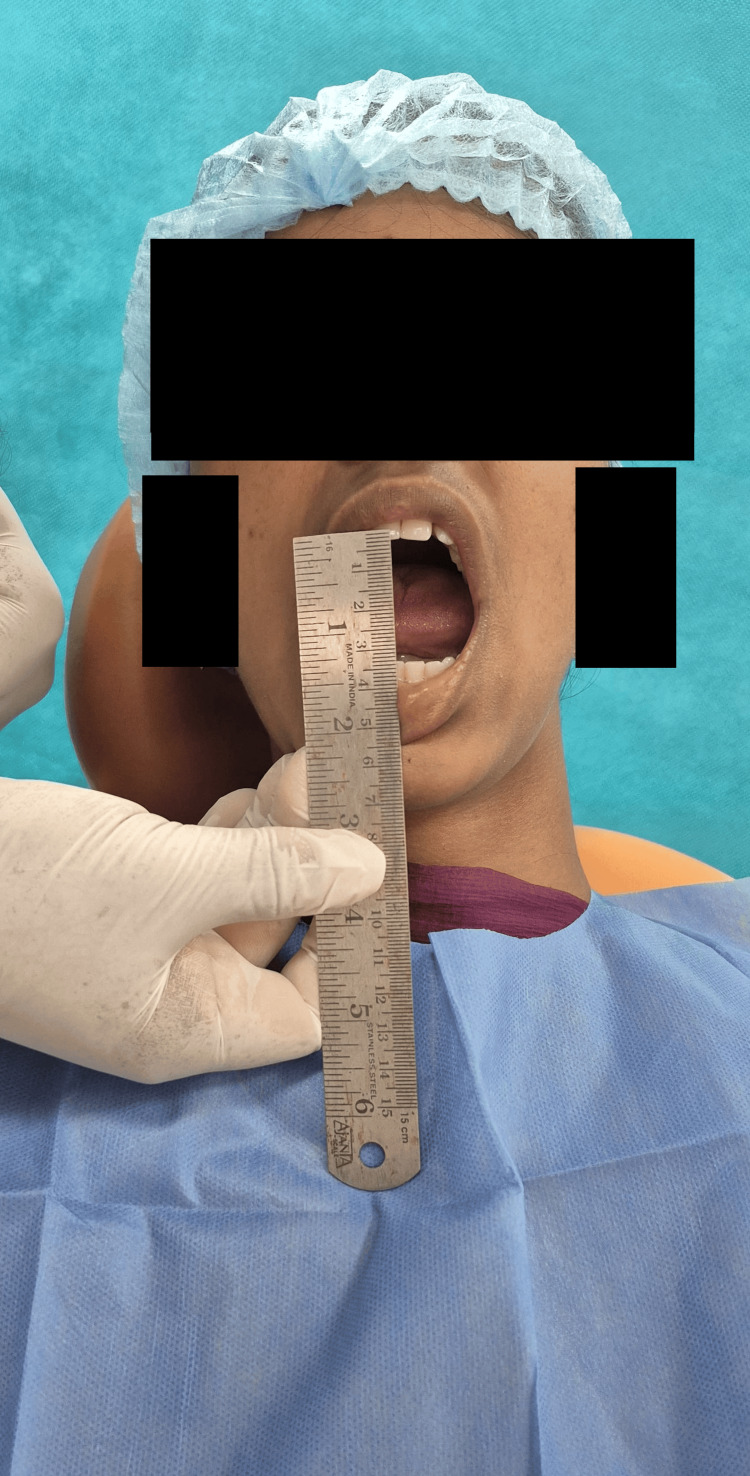
A pre-intervention mouth opening of 30 mm

**Figure 7:**
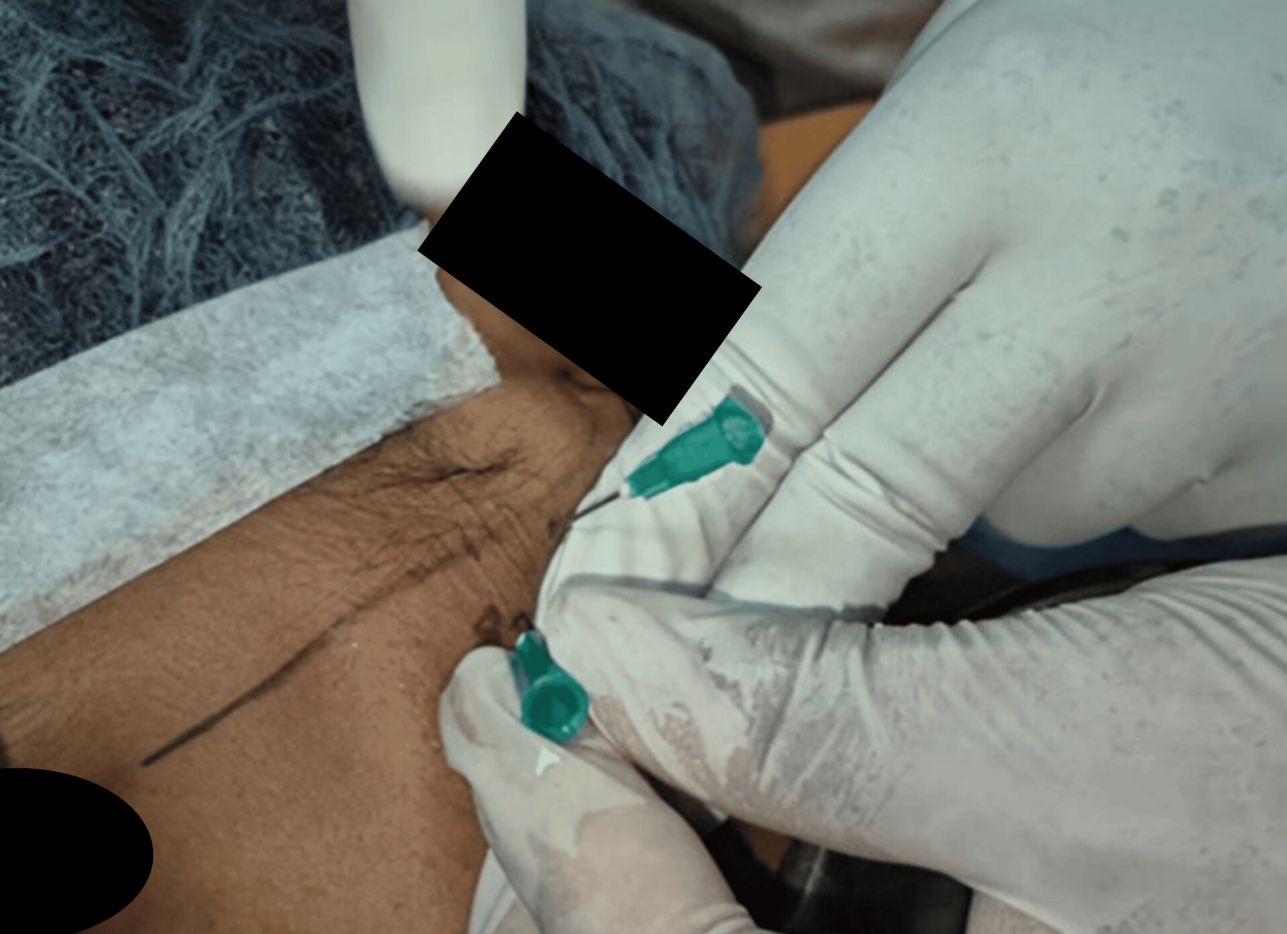
Needle inserted into Point A and Point B as per Nitzan’s procedure for arthrocentesis - two-needle technique

Another female patient aged 21 years presented to our department with a complaint of pain over the left TMJ and limited mouth opening for the past one month. Her pain got aggravated while chewing food and during speech. On examination, maximum mouth opening was 30 mm, as shown in Figure 6. The lateral movements of the TMJ were normal, and occlusion was stable. On palpation, tenderness was present over the left TMJ region, and on auscultation, clicking was present during opening and closing the mouth. MRI investigation was done, and it revealed a reducing articular disc during opening and closing the mouth, suggestive of Wilkes stage II classification of internal derangement of the right temporomandibular joint. The treatment plan was to manage her conservatively, followed by a minimally invasive regenerative procedure, and accordingly, she was advised a soft diet, hot fomentation over the left TMJ region, and to limit excessive mouth opening. Arthrocentesis was performed using a 2-needle technique (point A and point B) according to Nitzan guidelines using 100 ml of Ringer’s lactate solution, as shown in Figure 7. i-PRF was prepared from 10 ml of autologous blood collection and centrifugation at 700 rpm for 3 min. The obtained i-PRF was injected into the upper joint space of the TMJ through the needle in the point A position. The patient was evaluated for pain, joint sounds, mouth opening, functional limitations like chewing, yawning, speech, and patient satisfaction level at one week, one month, and three months post-intervention. All the values are tabulated as shown in Table 1.

Case 3

**Figure 8:**
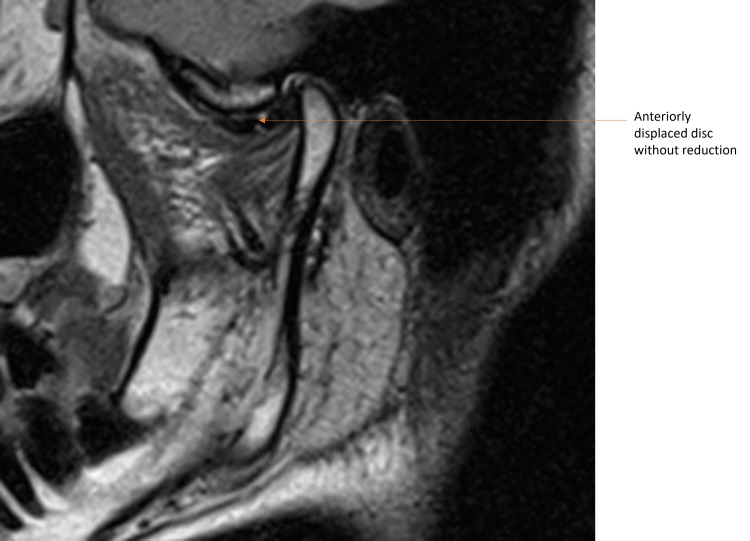
MRI scan (sagittal section) showing non-reducing anteriorly displaced disc during closing of the mouth

A male patient aged 34 years presented to our department with complaints of a clicking sound over the left side TMJ region while opening and closing the mouth for the past three months. On examination, maximum mouth opening was 38 mm, lateral movements of TMJ were normal, and occlusion was stable. On palpation, tenderness was present over the left TMJ region, and on auscultation, clicking was present during opening the mouth. An MRI investigation was done. Sagittal section of it revealed a non-reducing articular disc during closing the mouth, as shown in Figure 8, suggestive of Wilkes stage III classification of internal derangement of the right temporomandibular joint. The treatment plan was similar to case 2 reported above, where conservative management followed by a minimally invasive regenerative procedure was done, and accordingly, he was advised a soft diet, hot fomentation over the left TMJ region, and to limit excessive mouth opening. Arthrocentesis was performed using a 2-needle technique (point A and point B) according to Nitzan guidelines using 100 ml of Ringer’s lactate solution. i-PRF was prepared from 10 ml of autologous blood collection and centrifugation at 700 rpm for 3 min. The obtained i-PRF 1.5 ml was injected into the upper joint space of the TMJ through the needle in the point A position. Evaluation was done for pain, joint sounds, mouth opening, functional limitations like chewing, yawning, speech, and patient satisfaction level at one week, one month and three months post-intervention as shown in Table 1.

**Table 1:** Showing Pain, MMO, joint sounds, functional limitations, patient satisfaction at baseline, one week, one month and three months post-intervention MMO: Maximum Mouth Opening

Case/ Side	Age/sex	Wilkes stage	Time line	Pain VAS (0-10) Score	MMO	Joint sounds	Functional limitations			Patient Satisfaction (Likert’s scale)
							Chewing	Yawning	speech	
Case 1/ Left	42 years/F	III	Baseline	8	28mm	Clicking on opening	Difficult	Difficult	Difficult	Unsatisfied
			1 week	2	34mm	Normal	Difficult	Difficult	Normal	Satisfied
			1 month	0	40mm	Normal	Normal	Normal	Normal	Satisfied
			3 months	0	40mm	Normal	Normal	Normal	Normal	Satisfied
Case 2/ Left	21 years/F	II	Baseline	7	30mm	Clicking on opening and closing	Difficult	Difficult	Difficult	Unsatisfied
			1 week	2	35mm	Normal	Normal	Normal	Normal	Satisfied
			1 month	0	35mm	Normal	Normal	Normal	Normal	Satisfied
			3 months	0	38mm	Normal	Normal	Normal	Normal	Satisfied
Case 3/ Left	34 years/M	III	Baseline	2	38mm	Clicking on opening and closing	Difficult	Difficult	Normal	Unsatisfied
			1 week	0	38mm	Normal	Normal	Normal	Normal	Satisfied
			1 month	0	40mm	Normal	Normal	Normal	Normal	Satisfied
			3 months	0	40mm	Normal	Normal	Normal	Normal	Satisfied

## Discussion

Internal derangement of the temporomandibular joint (TMJ) is a common cause of orofacial pain and functional limitation, often associated with abnormal disc-condyle relationships. In 1978, Wilkes used clinical symptoms, surgical and radiological findings, and these findings were later combined with magnetic resonance imaging (MRI) findings of TMJ to define the criteria of TMJ ID and classify the same [12,13]. During the course of TMJ disorders, there are some unique "stages" that are manifested and can be defined and detected by MRI, representing the severity of the disease. These stages have clinical significance for prognosis and the treatment modality [14]. In the present case series, based on the clinical and MRI findings, two patients had anterior displacement of the disc without reduction, and one had anterior displacement of the disc with reduction.

Some of the salient signs and symptoms of ID of TMJ are clicking of the joint, pain during movements of the jaw, and limitation of the range of motion of the jaw. Understanding the signs and symptoms related to temporomandibular joint disorder and its direct impact on the patient’s quality of life makes the treatment of this disorder important [1,15]. Here also in the present case series, patients had joint pain, a clicking sound, and limitations in the jaw opening. Management of disorders of TMJ ranged from non-surgical approaches to surgical ones. Conservative therapy, as well as minimally invasive treatment, including physiotherapy, laser therapy, drug therapy such as muscle relaxants or NSAID's, occlusal splints, intra-articular injections, or a combination of these modalities, should be considered as a first-line approach before considering any invasive surgical procedures. Arthrocentesis of TMJ may act by allowing the elimination of hyper-viscous medium with catabolites and inflammatory cells, thereby counteracting the degeneration of tissues [16]. Some of the recent research supports certain therapies due to their positive effect, such as the injection of patients' own blood (autologous blood) or different platelet concentrates (PC) into the upper joint space of the temporomandibular joint in cases of TMJ-ID [17].

Injectable platelet-rich fibrin (i-PRF) is an autologous platelet concentrate prepared from peripheral blood using low-speed centrifugation (700 rpm for 3 minutes), resulting in a fibrin-rich matrix containing platelets, leukocytes, and bioactive growth factors. This fibrin scaffold acts as a biologic reservoir that allows the gradual and sustained release of growth factors, including platelet-derived growth factor (PDGF), transforming growth factor-beta (TGF-β), and vascular endothelial growth factor (VEGF) over several days to weeks, thereby enhancing tissue regeneration and repair [18,19].

PDGF plays a crucial role in tissue healing by stimulating the proliferation and migration of mesenchymal stem cells and fibroblasts, while promoting extracellular matrix (ECM) synthesis and collagen deposition. This supports fibrocartilage regeneration and structural repair of the temporomandibular joint (TMJ) [19]. TGF-β is essential for chondrogenesis and fibrocartilage formation within the articular disc. It regulates matrix remodeling, enhances synthesis of proteoglycans and collagen, and suppresses inflammatory responses, thereby preserving joint integrity and promoting disc regeneration [19]. VEGF contributes to neovascularization and angiogenesis, improving blood supply, oxygenation, and nutrient delivery to damaged tissues. Enhanced vascular support facilitates cellular survival, tissue remodeling, and regenerative healing within the TMJ microenvironment [17,19].

**Figure 9:**
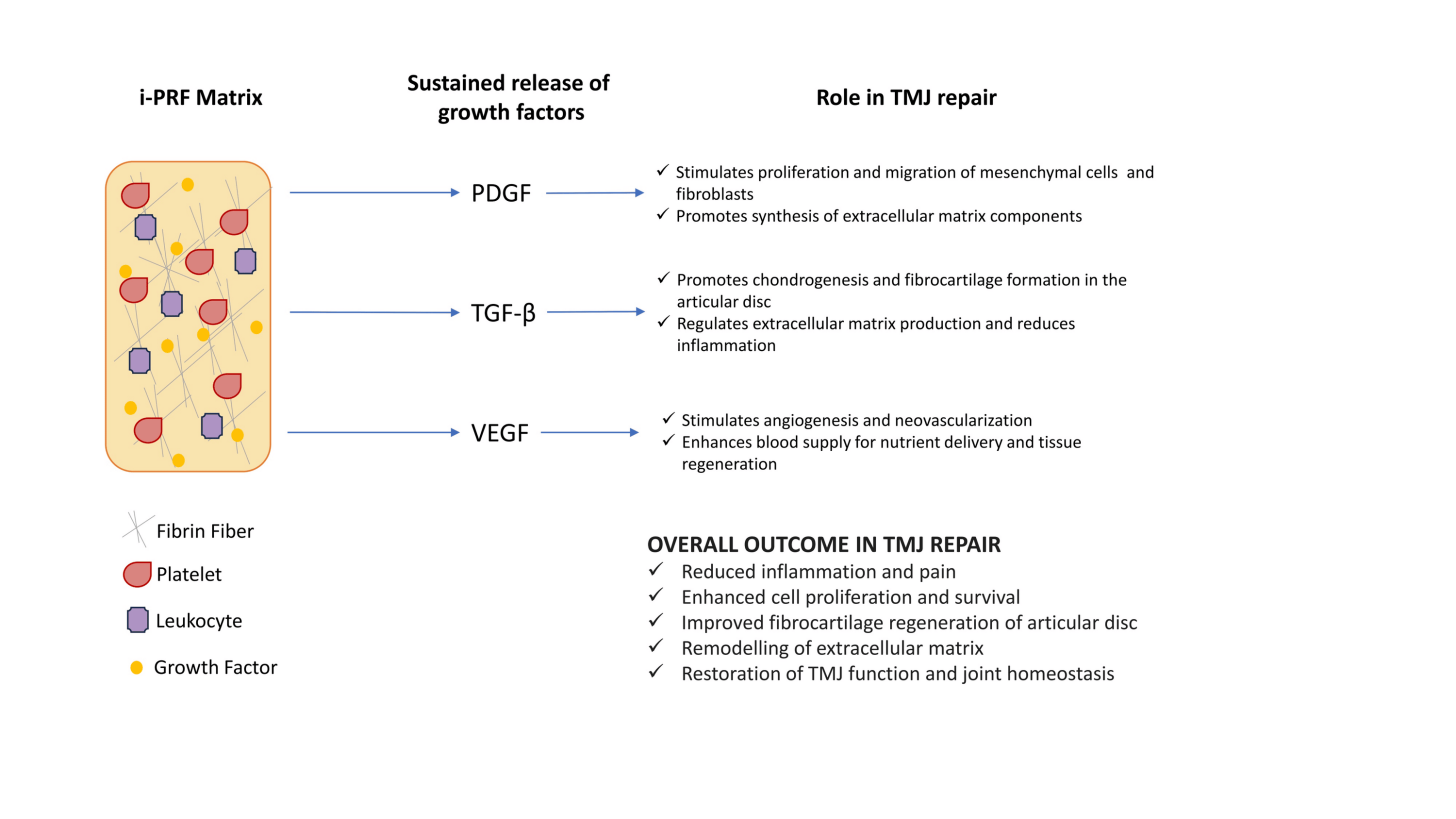
Pictorial representation of release of PDGF, TGF-beta and VEGF from i-PRF fibrin matrix and their roles in TMJ repair This image was created using Microsoft Office PowerPoint 2021, and no other generative AI image creation tool was used. PDGF: Platelet-derived growth factor; TGF-β: Transforming growth factor-beta; VEGF: Vascular endothelial growth factor; i-PRF: Injectable platelet-rich fibrin; TMJ: Temporomandibular joint

The synergistic action of these growth factors results in reduced inflammation and pain, enhanced cellular proliferation, improved extracellular matrix remodeling, regeneration of fibrocartilage, and restoration of TMJ homeostasis and function. Thus, i-PRF serves as a promising biologic adjunct for regenerative management of temporomandibular joint disorders, as shown in Figure 9 [18,19].

The present case series evaluated the clinical effectiveness of arthrocentesis of the TMJ followed by intra-articular injection of injectable platelet-rich fibrin (i-PRF) in the upper joint space, which was produced by centrifugation of 10 ml of autologous blood at 700 rpm for 3 minutes, in patients with different stages of TMJ internal derangement. The results demonstrated marked improvement in clinical parameters, including pain reduction, maximum mouth opening (MMO), joint sounds, and functional activities such as chewing, yawning, and speech in all the three cases. Pain was assessed using visual analogue scale (VAS 0 depicting no pain and 10 indicating maximum pain), showing a marked decrease as early as one week post-intervention, with complete resolution (VAS = 0) at one month and sustained up to three months in all cases. This rapid reduction in pain can be attributed to the combined effect of arthrocentesis in eliminating inflammatory mediators and i-PRF in modulating inflammation through the release of growth factors. Improvement in MMO was also notable across all cases. Case 1 (Wilkes stage III) showed an increase from 28 mm at baseline to 40 mm at one month, which was maintained at three months. Similarly, Cases 2 and 3 (Wilkes stage II & III, respectively) demonstrated progressive improvement in mouth opening. Arthrocentesis aids in releasing adhesions and improving joint mobility, while i-PRF may contribute to tissue healing and lubrication within the joint space, enhancing functional recovery. Joint sounds, particularly clicking, were present in all cases at baseline and resolved completely following intervention. This may be explained by improved disc-condyle coordination and reduction in intra-articular friction after lavage and biologic augmentation. Functional limitations, which were initially significant in Cases 1 and 2, improved progressively, with patients reporting normal function by one month. Even in Case 3, where baseline symptoms were milder, complete functional recovery was achieved early.

The outcomes observed in this case series are consistent with previous studies on arthrocentesis, which report considerable improvement in pain and mouth opening due to joint lavage and hydraulic distension. However, the addition of i-PRF introduces a regenerative component that may enhance and sustain these results. i-PRF releases bioactive molecules like platelet-derived growth factor (PDGF), transforming growth factor-beta (TGF-β), and also vascular endothelial growth factor (VEGF), which promotes angiogenesis, repair of injured tissues, and anti-inflammatory effects [19]. This could explain the sustained improvement observed over the three-month follow-up period. Another advantage of i-PRF is its autologous nature, ease of preparation, and minimal risk of adverse reactions. Compared to other intra-articular injectables such as corticosteroids or hyaluronic acid, i-PRF offers a biologically active and regenerative approach without the potential side effects associated with repeated steroid use. Notably, even in a more advanced case (Wilkes stage III), the combination therapy yielded favourable outcomes, suggesting that this approach may be effective across different stages of internal derangement. All the three patients were satisfied with the treatment outcome, which was assessed using the Likert scale. However, the degree of improvement and rate of recovery may vary depending on disease severity and duration. Despite these encouraging findings, this case series has certain limitations. Small sample size and short-term follow-up period limit the generalizability of the results obtained. Additionally, the absence of a control group makes it difficult to isolate the individual contribution of i-PRF from arthrocentesis alone. Long-term studies with larger sample sizes and randomized controlled designs are required to further validate the efficacy of i-PRF in TMJ disorders.

## Conclusions

Within the limitations of the case series presented here, the combination of arthrocentesis followed by intra-articular injection of i-PRF demonstrates a feasible, well-tolerated, and associated with a favorable short-term clinical outcome, warranting further controlled investigation. Also, it showed promising clinical outcomes in the management of temporomandibular joint (TMJ) internal derangement; all the patients showed good reduction in pain, improvement in maximum mouth opening, resolution of joint sounds, and restoration of functional activities such as chewing, yawning, and speech. These improvements were evident early and were sustained throughout the three-month follow-up period. Also, all the patients were satisfied with the treatment outcome. Since there is no arthrocentesis-only control arm, it is not possible to distinguish the contribution of i-PRF from the established effect of arthrocentesis alone. i-PRF's autologous nature, ease of preparation, and minimal risk profile make it a valuable adjunct in the minimally invasive management of TMJ disorders; however, in the present study, structural regenerative outcomes were not assessed. Further well-designed randomized controlled trials with larger sample sizes and longer follow-up periods are necessary to establish the definitive role of i-PRF in the treatment protocol of TMJ internal derangement.

## References

[1] Bahgat MM, Aly NM (2025). Is injectable platelet rich fibrin beneficial in managing intracapsular temporomandibular disorders? A systematic review and meta-analysis. BMC Oral Health.

[2] Satheesh G, Pratebha Balu, Bhagat JA, Sakthi S, Velmurugan P (2026). Intra-articular injections as a treatment option for internal derangement of the temporomandibular joint: a review. Asian J Dent Sci.

[3] Bahgat MM, EL-Helw NR, Abdelhamid AM, Fata MM, Ashour MS, Atteya A (2026). Intra-articular regenerative injection for management of painful irreducible temporomandibular disc displacement. Alexandria Dent J.

[4] Rahimov CR, Ali-Zada JK, Rahimov NR, Farzaliyev IM (2025). Magnetic resonance imaging for temporomandibular joint internal derangement and osteoarthritis - a prospective study. Ann Maxillofac Surg.

[5] Bertram S, Rudisch A, Innerhofer K, Pümpel E, Grubwieser G, Emshoff R (2001). Diagnosing TMJ internal derangement and osteoarthritis with magnetic resonance imaging. J Am Dent Assoc.

[6] Hegab AF, Al Hameed HI, Karam KS (2021). Classification of temporomandibular joint internal derangement based on magnetic resonance imaging and clinical findings of 435 patients contributing to a nonsurgical treatment protocol. Sci Rep.

[7] Kowalchuk RM, Kowalchuk RO, Kaplan-List K, Caplash JM, Block P (2018). Temporomandibular Joint Internal Derangement Score (TIDS): novel magnetic resonance imaging assessment score and its relation to invasive treatment in patients with clinical temporomandibular joint pathology. Heliyon.

[8] Young AL (2015). Internal derangements of the temporomandibular joint: a review of the anatomy, diagnosis, and management. J Indian Prosthodont Soc.

[9] Satheesh G, Balu P, Bhagat JA, Jagdish A, Selvakumar R (2026). Intra-articular injections for internal derangement of the temporomandibular joint: mechanisms, efficacy, and clinical perspectives. In: Medical Science: Updates and Prospects.

[10] Çiçek A, Güngörmüş M (2026). Evaluation of the efficacy of injectable platelet-rich fibrin in internal derangement of the temporomandibular joint: a retrospective study [Article in Press]. Int J Oral Maxillofac Surg.

[11] Hosgor H, Bas B, Celenk C (2017). A comparison of the outcomes of four minimally invasive treatment methods for anterior disc displacement of the temporomandibular joint. Int J Oral Maxillofac Surg.

[12] Warburton G (2021). Internal derangements of the temporomandibular joint. In: Oral and Maxillofacial Surgery for the Clinician.

[13] Wadhokar OC, Patil DS (2022). Current trends in the management of temporomandibular joint dysfunction: a review. Cureus.

[14] Gil C, Santos KC, Dutra ME, Kodaira SK, Oliveira JX (2012). MRI analysis of the relationship between bone changes in the temporomandibular joint and articular disc position in symptomatic patients. Dentomaxillofac Radiol.

[15] Bag AK, Gaddikeri S, Singhal A, Hardin S, Tran BD, Medina JA, Curé JK (2014). Imaging of the temporomandibular joint: an update. World J Radiol.

[16] Al-Hamed FS, Hijazi A, Gao Q, Badran Z, Tamimi F (2021). Platelet concentrate treatments for temporomandibular disorders: a systematic review and meta-analysis. JDR Clin Trans Res.

[17] De Riu G, Stimolo M, Meloni SM, Soma D, Pisano M, Sembronio S, Tullio A (2013). Arthrocentesis and temporomandibular joint disorders: clinical and radiological results of a prospective study. Int J Dent.

[18] Al-Delayme RM, Alnuamy SH, Hamid FT (2017). The efficacy of platelets rich plasma injection in the superior joint space of the tempromandibular joint guided by ultra sound in patients with non-reducing disk displacement. J Maxillofac Oral Surg.

[19] Miron RJ, Fujioka-Kobayashi M, Hernandez M, Kandalam U, Zhang Y, Ghanaati S, Choukroun J (2017). Injectable platelet rich fibrin (i-PRF): opportunities in regenerative dentistry?. Clin Oral Investig.

